# Comparison of double blastocyst transfer versus sequential transfer on pregnancy outcomes in individuals with frozen embryo transfer and a history of recurrent implantation failure: An RCT

**DOI:** 10.18502/ijrm.v23i4.18783

**Published:** 2025-06-10

**Authors:** Nooshin Hatamizadeh, Maryam Eftekhar, Zahra Aminimajomerd, Shahrzad Moeinaddini

**Affiliations:** Research and Clinical Center for Infertility, Yazd Reproductive Sciences Institute, Shahid Sadoughi University of Medical Sciences, Yazd, Iran.

**Keywords:** Assisted reproductive technology, Repeated implantation failure, Blastocyst transfer, Sequential embryo transfer, Pregnancy rates.

## Abstract

**Background:**

Recurrent implantation failure poses a significant challenge in assisted reproductive technology despite the transfer of high-quality embryos over multiple cycles.

**Objective:**

This study aimed to compare the clinical outcomes between double blastocyst transfer and sequential single cleavage-stage and blastocyst transfer in individuals undergoing frozen embryo transfer and those with a history of repeated implantation failure.

**Materials and Methods:**

This randomized clinical trial was conducted at the Yazd Research and Clinical Center for Infertility, Yazd, Iran from February to November 2024 and included 125 women ( < 45 yr) with a history of more than 2 implantation failures. Participants were randomized into 2 groups: one receiving double blastocyst transfer and the other receiving sequential single cleavage-stage and blastocyst transfer. The primary and secondary outcomes included clinical pregnancy, chemical pregnancy, early abortion, multiple pregnancy, and implantation rates.

**Results:**

Baseline characteristics were similar between the 2 groups. Chemical pregnancy rates were comparable (51.6% for double blastocyst transfer vs. 49.2% for sequential transfer, p = 0.790), as were clinical pregnancy rates (46.9% vs. 44.3%, p = 0.769). Early abortion rates showed no significant difference (27.3% vs. 20%, p = 0.498). Multiple pregnancy rates were similar (23.3% vs. 25.9%, p = 0.820), and implantation rates did not differ significantly (28.9% vs. 27.86%, p = 0.889).

**Conclusion:**

This study demonstrated that sequential single cleavage-stage and blastocyst transfer does not significantly improve assisted reproductive technology outcomes compared with double blastocyst transfer in individuals with recurrent implantation failure. Both methods had similar efficacy rates in terms of chemical pregnancy rates, clinical pregnancy rates, early abortion rates, multiple pregnancy rates, and implantation rates.

## 1. Introduction

Implantation success is a highly co-ordinated and complex process, which requires 2 essential components: a healthy, high-quality embryo and a receptive endometrium (1). The interaction between these 2 factors determines the likelihood of successful implantation, which is critical for achieving pregnancy in assisted reproductive technology (ART) cycles (2). Any disruption in this delicate balance can lead to implantation failure, which is a major challenge for individuals undergoing in vitro fertilization (IVF) treatments. Among these challenges, recurrent implantation failure (RIF) is particularly frustrating and poses a significant clinical problem for both individuals and clinicians (3). RIF is generally defined as the failure to achieve a clinical pregnancy despite the transfer of high-quality embryos over multiple IVF cycles (4). However, the exact definition of RIF varies across studies, leading to inconsistencies in its diagnosis and reporting. A systematic review addressed this issue and proposed that RIF can be defined as 2 consecutive failed frozen embryo transfer (FET) cycles (5). Despite attempts to standardize the definition, discrepancies persist, resulting in limited reliable data on the incidence and prevalence of RIF (6). This discrepancy inhibits RIF therapy development. Several methods have been tried to increase RIF implantation rates. Transferring blastocyst embryos is a potential therapy. Blastocyst transfer, which includes cultivating embryos until day 5, may improve embryonic selection and endometrial receptivity window synchronization. However, this method has hazards. Since not all embryos survive to day 5, blastocyst culture raises cycle cancellation risk (7). For this reason, a study has proposed using cleavage-stage embryo transfer (day 3) as an alternative to blastocyst transfer, as it reduces the risk of cycle cancelation and allows for earlier embryo placement into the uterus (8). Given the limitations of both cleavage-stage and blastocyst-stage transfers, sequential embryo transfer has emerged as a potential strategy to improve clinical outcomes, particularly in individuals with RIF. This approach aims to optimize endometrial receptivity by exposing the endometrium to embryos at different developmental stages, which may trigger beneficial endometrial changes, such as the release of cytokines and growth factors that promote implantation (9). In line with the findings of Homayoon et al. sequential embryo transfer may offer benefits beyond its established use in RIF, potentially extending to subpopulations such as patients with diminished ovarian reserve (10). Despite its theoretical advantages, the efficacy of sequential embryo transfer remains a topic of debate. While some studies have reported improved clinical outcomes and higher pregnancy rates with sequential transfer compared with single embryo transfers, others have found no significant differences in fertility outcomes between the 2 approaches (11). This discrepancy highlights the need for further research to clarify the role of sequential embryo transfer in improving implantation success. Currently, limited data are available regarding the clinical outcomes of sequential embryo transfer, and its effectiveness in individuals with RIF remains controversial (12). Given the challenges associated with RIF and the lack of consensus on optimal treatment strategies, additional studies are needed to evaluate the potential benefits of sequential embryo transfer. Therefore, this study aimed to assess the clinical outcomes of sequential embryo transfer, specifically the transfer of single cleavage-stage embryo to blastocyst-stage embryo, in individuals with RIF.

## 2. Materials and Methods

This randomized clinical trial with no blinding was conducted at the Yazd Research and Clinical Center for Infertility, Yazd, Iran between February and November 2024. Infertile women under 45 yr of age who presented to the center with a history of RIF (defined as more than 2 previous failures) and were candidates for FET were included in the study. All individuals undergoing a frozen-thawed cycle were treated with a protocol consisting of 6 mg of estradiol (Aburaihan Pharmaceutical Co., Tehran, Iran) daily, starting on day 2 of their menstrual cycle. On day 13 of the cycle, individuals were asked to visit the infertility center for an ultrasound evaluation.

Women with an endometrial thickness of 8 mm were prescribed progesterone, administered vaginally (400 mg progesterone suppository [Actogest, manufactured by Actoverco, Tehran, Iran] every 12 hr) and orally (10 mg dydrogesterone [Duphamed, manufactured by Actoverco, Tehran, Iran] every 12 hr). Women with severe uterine adenomyosis, severe Asherman's syndrome, uncontrolled autoimmune disorders (lupus and rheumatoid arthritis), uncontrolled endocrine disorders (diabetes, hyperthyroid, hypothyroid), egg donation, and untreated hydrosalpinx were excluded.

Eligible participants who had at least 4 frozen embryos available were then randomized into 2 groups: the sequential single cleavage-stage and blastocyst transfer group or the double blastocyst transfer group. In the double blastocyst group, embryo transfer was performed 5 days after the initiation of progesterone administration. In the sequential transfer group, a single cleavage-stage embryo was transferred 3 days after the start of progesterone, followed by a single blastocyst transfer 5 days after progesterone was initiated. Finally, in both groups, serum human chorionic gonadotropin (β-hCG) was measured 14 days after the embryo transfer (for the double blastocyst group) or 14 days after the second transfer (for the sequential transfer group). A β-hCG level of 50 IU/mL was considered positive for pregnancy, and luteal phase support was continued up to 12 wk of gestation.

### Baseline characteristics

The baseline characteristics of the participants, including age, body mass index, anti-Müllerian hormone levels, duration of infertility, type (primary or secondary), and causes of infertility (e.g., diminished ovarian reserve, male factor, polycystic ovarian syndrome, and unexplained infertility), were analyzed. These factors were assessed to ensure comparability and homogeneity between the study groups. Previous embryo transfer attempts, which are important variables influencing outcomes, were also evaluated for consistency across groups.

### Outcome measure

The primary outcome measure of this study was the clinical pregnancy rate, which was defined as the presence of a gestational sac with a fetal heartbeat confirmed via ultrasound 2 wk after a positive serum β-hCG test. Secondary outcomes included chemical pregnancy rate, which was defined as a serum β-hCG level ≥ 50 IU/mL measured 14 days after embryo transfer in the double blastocyst group or after the 2 ${}^{\text{nd}}$ transfer in the sequential transfer group.

Early miscarriage was defined as the loss of the gestational sac or the absence of a fetal heartbeat before the 10 ${}^{\text{th}}$ wk of pregnancy. The multiple pregnancy rate was determined using transvaginal ultrasound to identify the number of gestational sacs. The implantation rate was calculated as the ratio of gestational sacs observed on ultrasound to the total number of transferred embryos. Clinicians assessed all outcome measures and ultrasound evaluations.

### Sample size

The minimum sample size was estimated to be 70 in each group by considering a confidence interval of 95%, a power of 80%, 19% clinical pregnancy in the blastocyst transfer group, 40% difference, and considering a leakage of 10%. The sample size was determined using the study of Arefi et al. (13) and PASS15 software.

### Randomization procedure

Participants were randomly assigned to intervention or control group using a randomization list generated by Random Allocation software (version 1), produced by a statistical consultant. A statistical consultant informed the researcher of each participant's group assignment based on their entry number and the predetermined randomization list. This ensured that each participant was allocated to their respective study groups according to the randomized sequence.

### Ethical Considerations

The present study was approved by the Ethics Committee of Yazd Research and Clinical Center for Infertility, Shahid Sadoughi University of Medical Sciences, Yazd, Iran (Code: IR.SSU.RSI.REC.1402.020). The study was registered with the Iranian Registry of Clinical Trials on January 27, 2024 (IRCT20110509006420N27, updated on December 23, 2024). All participants provided written informed consent before enrollment. Participant data were anonymized to ensure confidentiality. Access to data were strictly limited to authorized personnel, and it was stored securely to prevent unauthorized access or disclosure. These measures were in accordance with the ethical guidelines of the Declaration of Helsinki and local regulations.

### Statistical Analysis

Statistical analyses were conducted using Statistical Package for the Social Sciences software version 26.0 (SPSS, Chicago, IL, USA). Continuous variables are reported as means ± SD, whereas categorical variables are presented as frequencies (percentages). The Mann-Whitney U test and independent *t* test were used to compare continuous variables between groups. In contrast, the Chi-square test and Fisher's exact test were employed to compare categorical variables. A 2-tailed p < 0.05 was considered statistically significant.

## 3. Results

**Figure 1:**
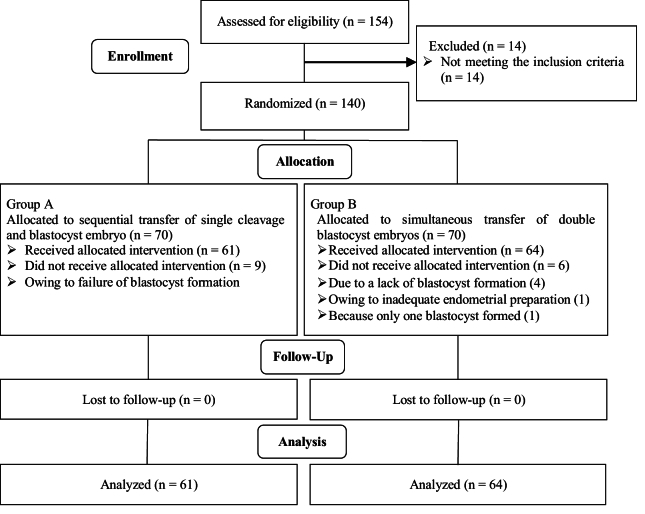
CONSORT flowchart.

A total of 125 individuals were included: 64 in the double blastocyst transfer group and 61 in the sequential single cleavage-stage and blastocyst transfer group. The study flowchart is presented in figure 1. The demographic and clinical features of the 2 study groups are summarized in table I. No significant differences in baseline traits were found, encompassing age, body mass index, anti-Müllerian hormone levels, duration of infertility, type of infertility (primary vs. secondary), causes of infertility, and the number of previous implantation failures. For detailed comparisons, see table I. Table II shows the outcomes of ART in 2 groups. The results indicate no statistically significant differences (p > 0.05) in various key pregnancy outcomes between the 2 groups. The rate of chemical pregnancies, as assessed using a positive β-hCG test, was similar between the double blastocyst transfer group (51.6%) and the sequential transfer group (49.2%), with no significant difference (p = 0.790). Clinical pregnancy rates, which indicate the presence of a gestational sac on ultrasound, were also comparable between the groups, with 46.9% in the double blastocyst transfer group and 44.3% in the sequential transfer group (p = 0.769). The incidence of early abortion, defined as miscarriage before 10 wk of gestation, did not differ between the groups. In the double blastocyst transfer group, 27.3% of chemical pregnancies resulted in early abortion, compared with 20% in the sequential transfer group (p = 0.498). The multiple pregnancy rates were also similar, with 23.3% in the double blastocyst transfer group and 25.9% in the sequential transfer group (p = 0.820). This indicates that the strategy of embryo transfer did not significantly affect the likelihood of multiple gestation. The implantation rate, defined as the ratio of detected gestational sacs to the number of embryos transplanted, was 28.9% in the double blastocyst transfer group and 27.86% in the sequential transfer group. The results did not significantly differ (p = 0.889).

**Table 1:** Demographic and clinical characteristics of the study groups

**Variables**		**One step of blastocyst transfer (n = 64)**	**Sequential cleavage and blastocyst transfer (n = 61)**	**P-value**
Age (yr)*		34.69 ± 5.61	34.51 ± 4.31	0.842
BMI (kg/m^2^)*		26.69 ± 4.01	26.08 ± 4.16	0.401
AMH (ng/Ml)**		4.39 ± 3.25 (3.50, 5.38)	4.72 ± 3.50 (3.30, 3.60)	0.614
Duration of infertility (yr)**		6.52 ± 4.40 (5.00, 6.75)	6.28 ± 3.96 (5.00, 6.00)	0.878
Infertility***				
	**Primary**	44 (68.8)	38 (62.3)	0.448
	**Secondary**	20 (31.3)	23 (37.7)	
Causes of infertility***				
	**DOR**	12 (18.8)	7 (11.5)	0.412
	**Male factor**	11 (17.2)	13 (21.3)	
	**Mix**	13 (20.3)	7 (11.5)	
	**Unexplained**	5 (7.8)	6 (9.8)	
	**PCOS**	21 (32.8)	21 (34.4)	
	**Tubal**	1 (1.6)	3 (4.9)	
	**Endometriosis**	1 (1.6)	4 (6.6)	
**Pervious embryo transfer failure (RIF)**				
	**< 3**	34 (53.1)	38 (62.3)	0.366
	**≥ 3**	30 (46.9)	23 (37.7)	
*Data presented as Mean ± SD, Student's *t* test. **Data are presented as Mean ± SD (Median, IQR), Mann-Whitney test. ***Data presented as n (%), Chi-square test. BMI: Body mass index, AMH: Anti-Mullerian hormone, DOR: Diminished ovarian reserve, PCOS: Polycystic ovarian syndrome, RIF: Recurrent implantation failure				

**Table 2:** ART outcomes between groups

**Variables**	**One step of blastocyst transfer (n = 64)**	**Sequential cleavage and blastocyst transfer (n = 61)**	**P-value**
Chemical pregnancy*	33 (51.6)	30 (49.2)	0.790
Clinical pregnancy*	30 (46.9)	27 (44.3)	0.769
Early abortion*	9/33 (27.3)	6/30 (20)	0.498
Multiple pregnancy*	7/30 (23.3)	7/27 (25.9)	0.820
Implantation rate**	37/128 (28.9)	34/122 (27.86)	0.889
Data presented as frequency (%). *Chi-square test, **Fisher's exact test. ART: Assisted reproductive technology			

## 4. Discussion

We compared double blastocyst transfer and sequential single cleavage-stage and blastocyst transfer in RIF patients. Chemical pregnancy, clinical pregnancy, implantation, early abortion, and multiple pregnancy rates were similar between groups. These findings support previous studies, whereas others show that sequential embryo transfer may be beneficial in some settings.

A research study found no significant differences in pregnancy outcomes between sequential embryo transfer (day 3 and day 5) and single blastocyst transfer on day 5 in people with 3 consecutive IVF failures. Clinical pregnancy rates were 40% vs. 38.3%, p = 0.85, similar to our study rates of 44.3% and 46.9% (p = 0.769) for sequential and double blastocyst groups (14). This suggests that sequential transfer does not universally outperform other methods when good-quality embryos are available. A study presented a nuanced view, showing that sequential transfer improved implantation (32.1%) and clinical pregnancy rates (50.7%) compared with double cleavage-stage transfer while matching double blastocyst transfer outcomes. They also noted lower rates of early miscarriage and multiple pregnancies in the sequential group (15). Conversely, our study did not find significant reductions in early abortion (20% vs. 27.3%, p = 0.498) or multiple pregnancy rates (25.9% vs. 23.3%, p = 0.820). These discrepancies could be attributed to variations in sample size, embryo quality, and participant characteristics.

A study further highlighted the benefits of sequential transfer, reporting higher rates of clinical pregnancies and live births compared with single-stage transfers. They suggested that sequential transfer might enhance embryo-endometrial interaction but cautioned that it requires sufficient good-quality embryos (12). Also, another study conducted a randomized controlled trial comparing sequential embryo transfer with double blastocyst transfer in intracytoplasmic sperm injection/FET cycles in individuals with RIF. Their results showed that sequential embryo transfer significantly increased implantation, clinical pregnancy, and ongoing pregnancy rates compared with double blastocyst transfer. Nonetheless, no significant differences were detected in the incidences of chemical pregnancy, multiple pregnancies, miscarriage, and ectopic pregnancy among the groups (16). A study conducted a systematic review to investigate the efficacy of sequential embryo transfer in IVF outcomes. The results demonstrated that sequential embryo transfer significantly improved clinical pregnancy rates compared with cleavage-stage transfer in both RIF individuals and non-RIF individuals. However, sequential embryo transfer did not provide significant benefits over blastocyst transfer. This systematic review supports the idea that sequential transfer may be particularly beneficial for improving clinical pregnancy rates compared with conventional cleavage-stage transfer (11).

A randomized controlled trial demonstrated that the sequential embryo transfer group had significantly higher clinical pregnancy, implantation, and ongoing pregnancy rates compared to the day 3 control group (12). These findings suggest that sequential embryo transfer may be particularly useful in settings where embryo freezing is not an option due to local regulatory restrictions.

A randomized controlled trial study evaluated the improvement of pregnancy rates in sequential (2-step) FET on day 3 or day 5 in individuals who experienced repeated IVF failures. The clinical pregnancy rates in the sequential FET group were significantly higher at 40% compared to 19% in the day 5 group (p < 0.001). This study highlights the effectiveness of the sequential transfer of frozen-thawed embryos on day 3 or day 5 compared with regular day 5 transfer in individuals with repeated IVF failure (13).

Our implantation rates were comparable between the sequential (27.86%) and double blastocyst (28.9%) groups, consistent with those of multiple studies that reported significantly higher implantation rates with sequential transfer, indicating that extended embryo exposure might optimize endometrial receptivity (14, 15). Another study also reported significantly higher implantation rates with sequential transfer, supporting the idea that sequential transfer may enhance endometrial receptivity (12). A study further reinforced the potential benefits of sequential transfer in improving implantation rates, particularly in individuals with repeated implantation failures (16). Another study also showed the effectiveness of sequential transfer in improving implantation rates in individuals with recurrent IVF failures (12).

Although a study reported improved clinical pregnancy rates with sequential transfer, our study did not. This may be due to our smaller sample size, which limits statistical power. The role of endometrial receptivity and embryo-endometrial crosstalk in sequential transfer requires further investigation (15). A systematic review further reinforced the potential benefits of sequential transfer in improving clinical pregnancy rates, particularly over cleavage-stage transfer (11). Another study also highlighted the significant improvement in clinical pregnancy rates with sequential transfer, especially in the context of regulatory restrictions on embryo freezing (12). Findings from prior research support the significant improvement in clinical pregnancy rates with sequential transfer in individuals with recurrent IVF failures. Multiple pregnancies pose significant risks for ART (13).

Our study found no significant difference in multiple pregnancy rates between the sequential and double blastocyst groups. In contrast, Gao et al. observed significantly lower rates of sequential transfer, possibly due to the transfer of single embryos at different stages. Early abortion rates were slightly lower in the sequential group (20%) than in the double blastocyst group (27.3%), although not significantly. Also, this study reported a significant reduction with sequential transfer (8.5% vs. 17.2%, p < 0.05), suggesting improved endometrial receptivity and embryo selection (15).

Our findings, along with those of other studies, suggest that sequential embryo transfer may benefit individuals with RIF, such as those with borderline endometrial receptivity or failed blastocyst culture. The success of this treatment depends on high-quality embryo availability and careful participant selection. For individuals with sufficient embryos, sequential transfer may be an alternative treatment, especially when the blastocyst culture failure risk is high. According to Madkour et al. sequential transfer can improve pregnancy outcomes in scenarios when embryo freezing is not possible. Sequential transfer may improve live birth rates and miscarriage rates, but large-scale, multicenter randomized controlled trials are needed (12). To improve RIF results, future research should examine endometrial receptivity indicators, genetic embryo screening, and advanced culture.

### Strengths and limitations

Our study's strengths include its randomized design and strict inclusion criteria, ensuring a homogenous RIF participant population with high-quality embryos. The small sample size may have limited our ability to detect subtle differences. Additionally, we did not evaluate ongoing pregnancy and live birth rates, which are crucial for assessing clinical benefits.

The present study has some limitations, including a single-center design, which may limit generalizability.

## 5. Conclusion

Our study showed that sequential single cleavage-stage and blastocyst transfer does not significantly improve ART outcomes compared with double blastocyst transfer in individuals with RIF. While some studies have suggested improved outcomes with sequential transfer, our findings indicate comparable efficacy. Embryo transfer strategies should be individualized based on participant-specific factors, embryo availability, and clinical circumstances. Further research is needed to clarify sequential transfer's role in improving RIF participant outcomes.

##  Data Availability

Data supporting the findings of this study are available upon reasonable request from the corresponding author.

##  Author Contributions

N. Hatamizadeh collected data and analyzed the data. M. Eftekhar designed and managed the study. Z. Aminimajomerd contributed to data collection. Sh. Moeinaddini contributed to data collection. All authors contributed to literature review, preparation of manuscript, and approval of the finished version.

##  Conflict of Interest

The authors declare that there is no conflict of interest.
